# Frontal eye field, where art thou? Anatomy, function, and non-invasive manipulation of frontal regions involved in eye movements and associated cognitive operations

**DOI:** 10.3389/fnint.2014.00066

**Published:** 2014-08-22

**Authors:** Marine Vernet, Romain Quentin, Lorena Chanes, Andres Mitsumasu, Antoni Valero-Cabré

**Affiliations:** ^1^Centre de Recherche de l'Institut du Cerveau et de la Moelle Epinière, CNRS UMR 7225, INSERM UMRS 975 and Université Pierre et Marie CurieParis, France; ^2^Laboratory for Cerebral Dynamics Plasticity and Rehabilitation, School of Medicine, Boston UniversityBoston, MA, USA; ^3^Cognitive Neuroscience and Information Technology Research Program, Open University of CataloniaBarcelona, Spain

**Keywords:** FEF, brain mapping, transcranial magnetic stimulation, visual performance, visuo-spatial attention, 3D vision, visual awareness, plasticity rehabilitation

## Abstract

The planning, control and execution of eye movements in 3D space relies on a distributed system of cortical and subcortical brain regions. Within this network, the Eye Fields have been described in animals as cortical regions in which electrical stimulation is able to trigger eye movements and influence their latency or accuracy. This review focuses on the Frontal Eye Field (FEF) a “hub” region located in Humans in the vicinity of the pre-central sulcus and the dorsal-most portion of the superior frontal sulcus. The straightforward localization of the FEF through electrical stimulation in animals is difficult to translate to the healthy human brain, particularly with non-invasive neuroimaging techniques. Hence, in the *first* part of this review, we describe attempts made to characterize the anatomical localization of this area in the human brain. The outcome of functional Magnetic Resonance Imaging (fMRI), Magneto-encephalography (MEG) and particularly, non-invasive mapping methods such a Transcranial Magnetic Stimulation (TMS) are described and the variability of FEF localization across individuals and mapping techniques are discussed. In the *second* part of this review, we will address the role of the FEF. We explore its involvement both in the physiology of fixation, saccade, pursuit, and vergence movements and in associated cognitive processes such as attentional orienting, visual awareness and perceptual modulation. Finally in the *third* part, we review recent evidence suggesting the high level of malleability and plasticity of these regions and associated networks to non-invasive stimulation. The exploratory, diagnostic, and therapeutic interest of such interventions for the modulation and improvement of perception in 3D space are discussed.

## Introduction: FEF, a crossroads for eye movements and visuo-spatial cognition

The frontal eye field (FEF) is an area of the frontal cortex in animals over which electrical stimulation is able to trigger eye movements. Electrophysiological studies in the monkey defined the FEF as an area containing visual, motor, and visuo-motor cells (Bruce and Goldberg, 1985) essential for the preparation and triggering of eye movements. This site operates as a crucial site of networks integrating other regions located in widespread locations. In humans for example, such gaze control systems include in the frontal lobe the supplementary eye field (SEF), the pre-supplementary eye field (pre-SEF), the dorsolateral prefrontal cortex (DLPFC), the cingulate eye field (CEF) within the anterior cingulate cortex and the dorso-medial frontal cortex, and in the parietal lobe, the parietal eye field (PEF) and areas of the posterior parietal cortex (PPC). Finally, subcortical structures, such as the superior colliculus (SC) in the midbrain are also considered essential to trigger eye movements. All these areas operate cooperatively, nonetheless some of them contribute to the triggering of eye movements under specific situations: the PEF for example has a role in reflexive saccades, the FEF participates in voluntary saccades, the SEF contributes to the development of more complex motor programs involving gaze (Pierrot-Deseilligny et al., 2002). Other areas, such as the CEF and the DLPFC, are more generally dedicated to cognitive aspects (e.g., motivation, memory) of oculomotor control (Gaymard et al., 1998b).

The anatomy of input and output projections within nodes of this network has been particularly well characterized in the monkey brain, and has revealed itself as a highly complex constellation of widespread interactions. The predominant neural inputs to the FEF originate in other cortical eye fields, including the SEF, the PEF, the middle superior temporal area, and the *principal sulcus* region (Schall et al., 1993; Tian and Lynch, 1996). The FEF also receives weak connections from the middle temporal area (MT), which may act as a relay between the striate / extrastriate cortices and the parietal cortex and FEF (Tian and Lynch, 1996). The FEF projects to many areas within the frontal cortex (Stanton et al., 1993), the occipital and parietal cortices such as V2/V3/V4, the middle temporal area (MT), the medial superior temporal area (MST) and the superior temporal visual area (Stanton et al., 1995). Finally, important reciprocal connections have been demonstrated between the FEF and the lateral intraparietal area (LIP) and more generally with the parietal cortex (Huerta et al., 1987; Cavada and Goldman-Rakic, 1989; Stanton et al., 1995; Tian and Lynch, 1996). Subcortically, the FEF projects directly to the brainstem (pons) (Leichnetz et al., 1984; Segraves, 1992). It also sends afferents to the SC (Schlag-Rey et al., 1992), either directly (Segraves and Goldberg, 1987) or indirectly *via* the basal ganglia (Stanton et al., 1988), and to other subcortical nuclei within the thalamus, subthalamus and tegmentum (Stanton et al., 1988). The FEF receives inputs from subcortical sites, including the substantia nigra, the SC. Finally, the cerebellum projects to thalamic regions innervating the FEF (Lynch et al., 1994).

Most of the earlier knowledge about the FEF was built-up on the basis of non-human primates experiments. A major emphasis has been put on the role of the FEF in the preparation and execution of saccades (Bizzi, 1968; Bruce and Goldberg, 1985). However, FEF also participates in the control of all the other types of eye movements, such as smooth pursuit or optokinetic nystagmus (OKN) (Bizzi, 1968; MacAvoy et al., 1991) and fixation (Izawa et al., 2004a,b, 2009). The intracortical stimulation of several subareas within the FEF is also able to trigger vergence movements (changes of the depth of the gaze) (Crosby et al., 1952, cited by Robinson and Fuchs, 1969). More recently, Gamlin and Yoon (2000) showed that a region within the pre-arcuate cortex in rhesus monkeys, immediately rostral to the saccade-related region in the anterior bank of the arcuate sulcus, is involved in vergence, accommodation and the sensorimotor transformations required for these movements. Moreover, Ferraina et al. (2000) showed that most neurons in a region of the anterior bank of the arcuate sulcus where saccades could be evoked with low current stimulation were also sensitive to disparity. The caudal portion of the FEF that contains smooth pursuit neurons also carries binocular signals related to vergence movement (Kurkin et al., 2003) and the majority of FEF pursuit neurons would respond to both frontal pursuit and pursuit in depth (Fukushima et al., 2002). There is also evidence that near and far spaces are differentially encoded in the frontal cortex including the FEF (Pigarev et al., 1979; Rizzolatti et al., 1983). Thus, the FEF appears to be involved in every sort of eye movements in 3D space.

It is expected that human FEF will be recruited, as in animals, for all types of ocular behavior: saccades, fixation, smooth pursuit, OKN, vergence. However, within each type, the specific experimental set-ups conditioning different categories of eye movements (e.g., reflexive, voluntary) will modulate the involvement of the FEF. Indeed, different cortical oculomotor areas are differentially recruited according to the category of eye movements (Gaymard et al., 1998a). The fact that the cognitive context is modulating the involvement of the FEF is reminiscent of the other roles played by the FEF in visuo-spatial attention, visual awareness, and perceptual modulation.

Before entering into the details on the various roles of the FEF (part II) and how its activity can be modulated for clinical purposes (part III), we will describe the efforts made to localize this area in both non-human and human primates (part I). As expected from an area contributing to numerous functions, the exact localization will strongly depend on the methods and specific paradigms used to assess it.

## Localization of FEF

The primate FEF is defined physiologically as the portion of the dorsolateral prefrontal cortex from which low-intensity intracortical stimulation is able to elicit rapid eye movements. Using this invasive approach, the monkey FEF has been located by some studies in the frontal lobe along the anterior border of the arcuate fissure, which would correspond to Brodmann's area 8, or overlapping with both areas 8 and 6 (or, using Walker's nomenclature, with areas 8A and 45) (for a review, see Tehovnik et al., 2000). According to the results of neuroimaging studies, the human FEF is mostly thought to be located in the superior pre-central sulcus near the caudal end of the superior frontal sulcus, which corresponds to Brodmann's area 6. However, as will be described in the second part of this review, the FEF contributes not only to several aspects of eye movements but also to different cognitive domains, and the exact location of the FEF will strongly depend not only on the methods (e.g., stimulation vs. neuroimaging) but also on the tasks (e.g., type of eye movements and type of control conditions, see e.g., Paus, 1996) and activation criteria (e.g., intensity of stimulation, see Blanke et al., 2000 for a discussion) used. Overall, it is still not entirely clear whether the reported inter-species differences in FEF location can be related to genuine anatomical differences between non-human primates and humans, caused by the use of different mapping methods or they simply reflect interindividual differences, which have not always been systematically studied in large cohorts of animals and human participants.

In the next pages, we will review some of the numerous studies that have attempted to determine the anatomical location of the FEF employing: microstimulation, intracranial recordings, functional magnetic resonance imaging (fMRI), positron emission tomography (PET), magnetoencephalography (MEG) and transcranial magnetic stimulation (TMS). A summary of the localizations reported in these studies can be found in Table 1.

**Table 1:** **Localization of FEF across studies, techniques and species**.

**Technique**	**Localization**	**Studies**
Microstimulation and recordings in non-human primates	Posterior part of the pre-arcuate sulcus	Bizzi, 1968; Robinson and Fuchs, 1969; Wurtz and Mohler, 1976; Bruce and Goldberg, 1985; Bruce et al., 1985; Segraves and Goldberg, 1987; MacAvoy et al., 1991; Gottlieb et al., 1993, 1994; Izawa et al., 2004a,b, 2009
	stimulation of the dorsal premotor area in owl monkeys can also evoke saccades	Preuss et al., 1996
Microstimulation in implanted patients	Posterior part of the middle frontal gyrus	Foerster, 1936
	All frontal gyri and pre-central gyrus	Rasmussen and Penfield, 1948
	At the level of and in front of the motor representation	Godoy et al., 1990
	Posterior part of the middle frontal gyrus and neighboring portions of the superior frontal gyrus but not in the inferior frontal gyrus or in the pre-central sulcus	Blanke et al., 2000
PET	Anterior portion of the pre-central gyrus	Fox et al., 1985; Anderson et al., 1994; Law et al., 1997
	Posterior portion of the pre-central gyrus	Sweeney et al., 1996
	Pre-central sulcus	Petit et al., 1995, 1996; Paus, 1996
	Middle frontal gyrus (about 3.5 cm anterior to the precentral sulcus and 1.1 cm posterior to the DLPFC)	Kawashima et al., 1998; Interpretation by Tehovnik et al., 2000
fMRI	Several foci within the pre-central sulcus, at the junction of the superior frontal sulcus, potentially extending to the pre-central gyrus	Darby et al., 1996; Muri et al., 1996; Petit and Haxby, 1999; Petit et al., 1997; Berman et al., 1999; Luna et al., 1998; Corbetta et al., 1998; Beauchamp et al., 2001; Rosano et al., 2002; Grosbras et al., 2005
	Pre-central sulcus, at the junction of the middle frontal gyrus	Amiez et al., 2006
fMRI in non-human primates	3 foci: 1 in the bank of the arcuate sulcus, and 2 in the inferior and superior precentral sulci	Koyama et al., 2004
MEG	Rostral location; or shift from the rostral (similar to microstimulation non-human primates studies) to the caudal (similar to human neuroimaging studies) location during saccade preparation	Ioannides et al., 2004, 2005, 2010
TMS	2 cm anterior to the inter-aural line, approximately 6 cm lateral to the vertex, between areas over which TMS evokes motor potential in hand's and face's muscles (or possibly more rostrally)	Thickbroom et al., 1996
	2 or 1.5 cm rostral to the motor hand area (probably belonging to the middle frontal gyrus close to the pre-central sulcus)	Ro et al., 1999, 2002
	FEF determined anatomically (within the middle frontal gyrus, rostral from the junction of the pre-central and the superior central sulci), then the authors measured that this area was about 3–4 cm rostral to the motor hand area representation; Talairach coordinates close to the ones from Paus (1996)	O'Shea et al., 2004; Silvanto et al., 2006

### Microstimulation and recordings in non-human primates: the original definition

In 1874, Ferrier summarized stimulation studies performed on several animal species including cats, dogs and rabbits as follows: “*In the superior frontal convolution, in advance of the centre for certain forward movements of the arm, as well as in the corresponding part of the middle frontal convolution, are areas, stimulation of which causes lateral (crossed) movements of the head and eyes and dilatation of the pupils.”* (Ferrier, 1874).

More than one hundred years later, other microstimulation studies evoking eye movements (Robinson and Fuchs, 1969; MacAvoy et al., 1991; Gottlieb et al., 1993; Izawa et al., 2004a,b, 2009), electrophysiological recordings during visual stimulation and/or eye movements (Bizzi, 1968; Wurtz and Mohler, 1976; Bruce and Goldberg, 1985; Segraves and Goldberg, 1987) and studies comparing cells discharge patterns during behavior or its alteration during the stimulation of these same neuronal populations (Bruce et al., 1985; Gottlieb et al., 1994) confirmed the existence of an FEF located in the posterior part of the pre-arcuate sulcus. They distinguished visual (modulated by functional significance), motor and visuo-motor neural populations for saccade, pursuit and fixation/saccade suppression, somehow spatially segregated and with different stimulation thresholds, which depended on the activation state of the monkey at the time of the stimulation. Each sub-region showed its specific organization. For saccades for instance, stimulation of ventro-lateral regions evoked small amplitude saccade whereas stimulation of dorso-medial regions induced large saccades; moreover the direction of the saccades varied as a function of the depth of stimulation in the arcuate sulcus (Tehovnik et al., 2000). Interestingly there is also evidence that in some primate species (e.g., owl monkeys), the stimulation of the dorsal premotor area, posterior to the usually defined FEF, can also evoke saccades, suggesting that such posterior area, potentially closer to the human FEF, could also belong to the non-human primates FEF (Preuss et al., 1996).

Although microstimulation is considered a gold standard technique to reveal a causal relation between a region and a brain function, it has potential limitations (see Amiez and Petrides, 2009; for review). First, the extent and number of responding areas depends on stimulation intensity, whose traditional threshold level (50 μ) is set up arbitrarily. Second, within the same study or across studies and depending on the experimental design chosen, some cortical areas have been less systematically sampled than others, a fact that could have biased output maps overemphasizing the role of certain locations while undermining the contribution of others. Third and last, intracortical stimulation can evoke eye movements from direct FEF activation, but also by activating intracortical white matter pathways connecting the FEF to other areas (Luna et al., 1998), a phenomenon that could easily blur the borders of cortical representations and lead to mislocalizations.

### Microstimulation in humans

Microstimulation procedures have not been solely restricted to a use in animal models. They have also been occasionally performed in epileptic patients, either per-operatively or outside of surgery rooms in more ecological conditions via chronically implanted subdural electrodes in fully awake patients. Using the first procedure, Foerster (1936, cited by Blanke et al., 2000) induced eye movements only from the posterior part of the middle frontal gyrus, whereas Rasmussen and Penfield (1948 cited by Blanke et al., 2000) report to have induced similar effects from all frontal gyri and the pre-central gyrus. With implanted subdural electrodes at the level of and in front of the motor representation, Godoy et al. (1990) evoked contralateral conjugate eye movements (mostly saccades), and sometimes accompanying head version following eye deviation. Blanke et al. (2000) investigated systematically the current intensity needed to elicit unilateral eye movements and found, consistently with monkey studies, that the eye fields inducing saccades and smooth eye movements are located in the posterior part of the middle frontal gyrus and neighboring portions of the superior frontal gyrus but not in the inferior frontal gyrus or in the pre-central sulcus.

Thus, microstimulation in well-controlled settings in human patients can yield results equivalent to those demonstrated in non-human primates with similar interventions. As also mentioned above for the animal, the intensity used for intracortical stimulation in humans arbitrarily determines the number and the size of the cortical clusters that activated directly or indirectly by connectivity are ultimately causally associated to the FEF. In addition, such studies are also constrained by the spatial location, distribution, and coverage of the implanted electrodes, which are strictly guided on the basis of clinical and not scientific criteria, and limited by the scarcity of time available for testing and the lack of large cohort of similarly implanted patients available to provide statistical evidence. Moreover, for ethical reasons, such procedures are only performed in human patients who have undergone developmental or acquired anatomical and functional alterations and do not necessarily provide accurate information on the healthy brain. In view of such limitations, non-invasive neuroimaging techniques, such as PET, fMRI, MEG and also non-invasive neurostimulation by TMS have become particularly popular in cognitive neuroanatomy and have been employed in the quest to locate the FEF in both humans and to a lesser extent in animals.

### Neuroimaging

The spreading of neuroimaging techniques such as PET and more recently fMRI has allowed the evaluation of FEF location and function in healthy human brains. The gradual increase of spatial resolution has permitted defining progressively smaller and better-delimited regions corresponding to the FEF. Within the large FEF region characterized by means of PET, several subareas associated with eye movements have been revealed using fMRI.

The variability of FEF location and function, found across different PET studies, has been reviewed by Paus (1996). Pioneering explorations using PET reported large activations in the human lateral frontal cortex during saccade execution. Most of these studies defined the FEF as part of the pre-central sulcus in the frontal lobe (Petit et al., 1995, 1996). Nonetheless this region has been sometimes localized in the anterior portion of the pre-central gyrus around the pre-central sulcus (Fox et al., 1985; Anderson et al., 1994; Law et al., 1997), or within the posterior portion of the pre-central gyrus around the central sulcus (Sweeney et al., 1996). A large range of eye movement types have shown to activate the FEF: fixation (Petit et al., 1995), reflexive or memory saccades (Anderson et al., 1994), saccades with or without visual cues (Fox et al., 1985), suppressed or imagined saccades (Law et al., 1997), anti-saccades (O'Driscoll et al., 1995; Sweeney et al., 1996), predictive saccades and gaze pursuit (O'Driscoll et al., 2000). Some of these studies showed that the intensity of FEF activation was neither influenced by target presence, cue type, task complexity (Fox et al., 1985) nor by whether the saccades were voluntary or previously learned (Petit et al., 1996), whereas other studies showed, on the contrary, a modulation of FEF activation from fixation to reflexive or volitional saccades (O'Driscoll et al., 1995; Sweeney et al., 1996).

The higher spatial resolution of fMRI recordings in humans has allowed researchers to restrict the site hosting the FEF along the pre-central sulcus (Darby et al., 1996; Muri et al., 1996). It also permitted to identify within this sulcus, several sub-areas subtending potentially distinct functions related to saccadic activity. Petit and Haxby (1999) and Petit et al. (1997) reported the FEF as located at the junction of the pre-central sulcus and the superior frontal sulcus extending laterally to the pre-central gyrus. They described a saccade-related FEF and a smaller, more inferior, and more lateral gaze pursuit-related FEF, which according to another study could overlap (Berman et al., 1999). Rosano et al. (2002) found a restricted area within the pre-central sulcus, integrating the saccade area, as located mainly on the rostral bank close to the cortical surface, and the pursuit area situated deeper in the sulcus, suggesting similar superficial/deep activation as the one characterizing non-human primates. Activation restricted to the pre-central sulcus was also shown in individual subjects in the study from Luna et al. (1998) contrasting simple visually-guided saccades to fixation. They described a consistent activation of the superior portion of the pre-central sulcus and a less consistent activation of the inferior portion of the pre-central sulcus. Similarly, different clusters of activation within the pre-central sulcus were found in other studies (Corbetta et al., 1998; Beauchamp et al., 2001). A meta-analysis performed on PET and fMRI datasets confirmed that for both visually and voluntarily-triggered saccades, the FEF lies in the pre-central sulcus close to its intersection with the superior frontal sulcus, potentially extending onto the superior and inferior subregions of the superficial portions of the pre-central gyrus (Grosbras et al., 2005). It should be noted, however, that some recent studies localized the superior FEF within the ventral portion of the superior pre-central sulcus, either at the end or at the most posterior region of the middle frontal gyrus, instead of at the level of its intersection with the superior frontal sulcus (Amiez et al., 2006).

Neuroimaging approaches show some limitations as compared to neurostimulation methods to determine the brain regions involved in a given saccadic behavior. First, neuroimaging methods are less sensitive than neurostimulation approaches in the detection of small saccade-related areas (Luna et al., 1998); second, group-averaging strategies employed in neuroimaging approaches to increase statistical power may come at the risk of shifting activation sites in case of strong interindividual anatomical differences (Luna et al., 1998; Amiez and Petrides, 2009); third, whereas brain stimulation mostly reveals contralaterally-evoked saccades, fMRI studies are built on protocols embedding bilateral and repetitive eye movements conditions and compared to a gaze fixation baseline. Hence differences in region size and shifted FEF localizations (Blanke et al., 2000) could be well caused by either the influence in contrast analyses from cells within the FEF involved in fixation and/or the mix up of activity related to different saccade directions within the same analyses. Another important concern raised by Tehovnik et al. (2000) and Amiez and Petrides (2009) is that in neuroimaging protocols, no instruction is given regarding blinking and return-to-center saccades in between trials (which is often accompanied by blinks). This could also explain rather posterior mislocalizations of the FEF, which would mistakenly encompass activity from regions within the motor strip involved in eyelid motion. In favor of this possibility, a PET protocol with multiple saccades, inducing comparable blinks frequency in the saccade and the control condition, found activity within the middle frontal gyrus (Kawashima et al., 1998). This observation is consistent with a more anterior location for the FEF in the frontal lobe (Tehovnik et al., 2000) and argues in favor of important blinking-related biases in prior PET and fMRI explorations.

**Figure 1:**
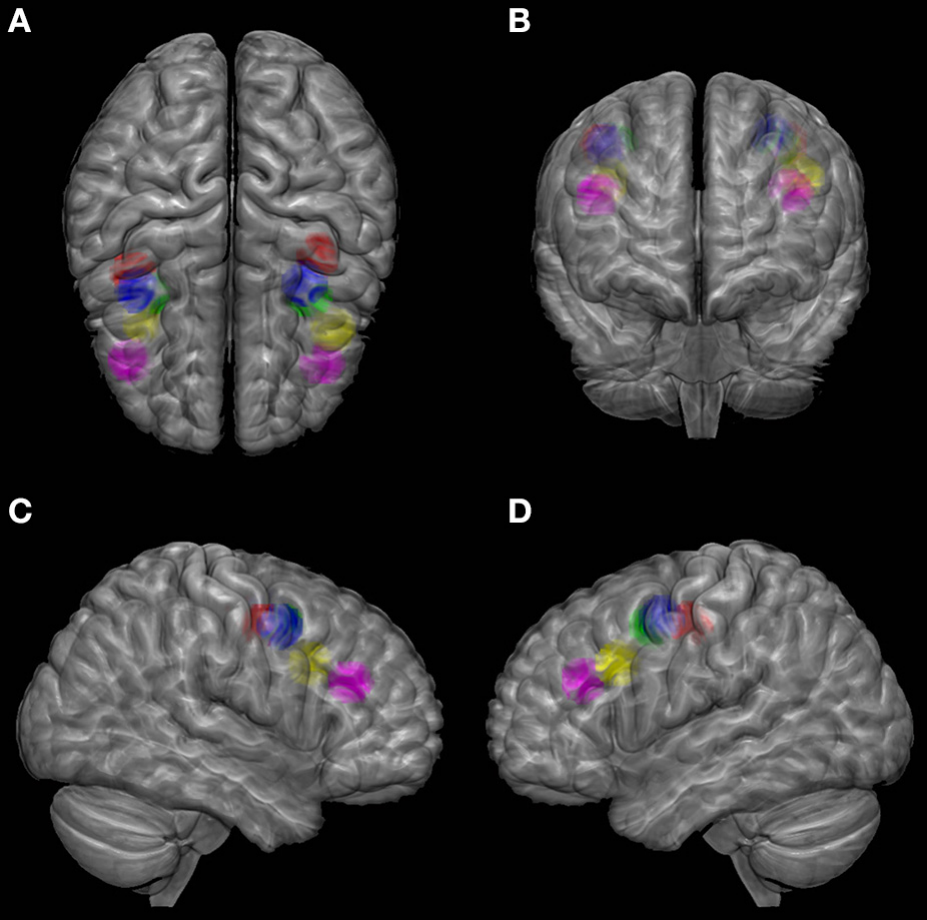
**Localization of FEF according to several studies on the MNI (Montreal Neurological Institute) brain template viewed from top (A), front (B), right (C) and left (D)**. Color codes as follows. Green: meta-analysis of PET studies from Paus (1996); Blue: fMRI study of Luna et al. (1998); Red: fMRI study of Petit and Haxby (1999); Yellow: MEG study of Ioannides et al. (2004); Purple: coordinates estimated by Tehovnik et al. (2000) based on the PET study of Kawashima et al. (1998). A sphere of 1 cm radius is positioned at the center of FEF activation from each study. SPM (Statistical Parametric Mapping, http://www.fil.ion.ucl.ac.uk/spm/) with MarsBar toolbox was used to design the spheres and MRIcroGL software (http://www.mccauslandcenter.sc.edu/mricrogl/) was used for glass brain illustration.

In spite of the above-mentioned problems, neuroimaging studies still have the advantage of providing normalized coordinates corresponding to group mean activation peaks (see Figure 1) that can be easily compared across studies and used as targets for subsequent non-invasive brain stimulation approaches on search of causality. In that vein, the meta-analysis of 8 PET studies involving 62 healthy participants designed by (Paus, 1996) suggested a reference location in Talairach coordinates (Table 2). Subsequent fMRI have contributed Tailarach coordinates reflecting similar or more posterior loci for the main (e.g. the superior) FEF site during the active performance of saccades or, on the contrary, more anterior location when blinks were avoided (Table 2).

**Table 2:** **Coordinates of left and right FEF from a few neuroimaging studies**.

**Study**	**Method**	**Number of subjects**	**Talairach coordinates left FEF [X; Y; Z]**	**Talairach coordinates right FEF [X; Y; Z]**
Paus, 1996	PET	*N* = 62 (meta-analysis of 8 studies)	[−32 ± 11; −2 ± 4; 46 ± 4]	[31 ± 11; −2 ± 5; 47 ± 5]
Petit and Haxby, 1999	fMRI	*N* = 5	[−35 ± 4; −18 ± 5; 46 ± 1]	[36 ± 5; −10 ± 4; 47 ± 3]
Luna et al., 1998	fMRI	*N* = 10	[−30 ± 7; −7 ± 7; 49 ± 7]	[34 ± 9; −3 ± 5; 47 ± 5]
Data from Kawashima et al. (1998), estimation & interpretation from Tehovnik et al. (2000)	PET; study that happen to avoid blinks	*N* = 9	[−37 ± 5; 26 ± 12; 29 ± 8]	[37 ± 5; 26 ± 12; 29 ± 8]
Ioannides et al., 2004	MEG	*N* = 3	[−41 ± 7; 12 ± 8; 34 ± 12]	[32 ± 7; 10 ± 14; 34 ± 7]

### Reconciling non-human primates' and humans' locations for the FEF?

In brief, the non-human primate FEF, localized mainly thanks to microstimulation studies, lies in a more rostral location (Brodmann's area 8) than the human FEF, localized mainly thanks to neuroimaging studies (Brodmann's area 6). A suggestion to reconcile such discrepancies between monkey and human reports is that the more posterior FEF location in humans has been erroneously attributed to Brodmann's area 6. Following that line, a study focused on the delimitation of cytoarchitectonics areas in post-mortem human brains containing the superior element of the pre-central sulcus and the caudal end of the superior frontal sulcus (Rosano et al., 2003). This study suggested that the pre-central sulcus might represent a transitional area between the rostral granular cortex and the caudal agranular cortex. Thus, the FEF would be located within a region that appears to have a similar chemoarchitecture (Stanton et al., 1989; Rosano et al., 2003) in both species, even if lying in a more caudal location in humans.

Other studies have suggested that discrepancies between monkeys and humans in FEF location arise from methodological differences rather than from a genuine inter-specie divergence. We already mentioned that microstimulation in humans can yield results equivalent to those demonstrated in non-human primates with similar interventions (see Section “Microstimulation in Humans). Do monkey fMRI recordings also reveal similar activations than the ones shown in humans with this same mapping technique? Koyama et al. (2004) conducted an fMRI study in macaque monkeys and revealed three saccade-related foci of activation. One was located in the bank of the arcuate sulcus, approximately in Brodmann's area 8, which corresponds to the classical non-human primate FEF, whereas the remaining two laid in premotor areas, and more precisely, in the inferior and superior precentral sulci within Brodmann's area 6. Thus, monkey fMRI studies reveal indeed activations similar to those found in humans. Further studies are needed to conclude on whether the discrepancy between non-human primates and humans results mainly arises from different cytoarchitectonics areas in different species or from the use of different methods. Probably, a deeper exploration of the multiple foci associated with the FEF will help to clarify its role and localization across species.

Finally, the use of a third methodology can shed a new light on the interpretation of results arising from monkeys' microstimulation and humans' fMRI studies. Taking advantage of the exquisite temporal resolution of MEG and the possibility of localizing source signals with a reasonable spatial resolution Ioannides et al. (2004, 2005) suggested an anterior location similar to the one found in microstimulation studies (e.g., in Ioannides et al., 2004 in 3 subjects, see Figure 1 and Table 2 for Talairach coordinates; however, note the high inter-individual variability of the Y coordinate between 24 and −3 for the right FEF). According to a MEG single subject study of this same group, the activity associated to the FEF could shift along a rostro-caudal axis, from the rostral site identified in microstimulation studies to the caudal region reported in fMRI studies, during the saccade preparation time (Ioannides et al., 2010), suggesting an unexpected confounding role of this variable. Most importantly, this study suggested that both the rostral (usually described for the non-human primates) and the caudal (usually described for the humans) sites can be identified in humans at different timing.

### TMS: in search of a causal functional localizer in healthy humans

In order to overcome the limitations of invasive human microstimulation but still benefit from its causation power, some researchers have turned to TMS as a causal brain mapping technique. TMS is based on a non-invasive induction of small currents intracortically in order to modulate brain activity at specific cortical areas with a relatively good spatial resolution, in the order of 1.2–3.5 mm radius (Wagner et al., 2007; Bijsterbosch et al., 2012). Depending on variables such as the stimulated area, magnetic pulse intensity, pre or post event time window chosen for pulse delivery, or the temporal distribution of individual pulses employed either in short bursts or long stimulation patterns, TMS can have an immediate (i.e., the so-called *online*) or lasting (so-called *offline*) facilitatory or disruptive impact on neurophysiological activity and consequently on the performance driven by the targeted cortical region and its associated network of areas (Valero-Cabre et al., 2005, 2007). Thanks to these properties, this technique is used to explore the causal contribution of different cortical areas and associated anatomical systems to human behavior in healthy individuals, whereas in clinical applications TMS has been employed to manipulate patterns of activity and drive therapeutically interesting outcomes for neurological or neuropsychiatric conditions (Valero-Cabre et al., 2011).

As TMS operates by using a magnetic field to non-invasively induce electrical current within the cortex, it has been hypothesized that, as intracranial electrical stimulation does, magnetic stimulation should also be able to trigger eye movements. However, TMS delivered systematically into frontal locations where the FEF is located has surprisingly proven unable to trigger eye movements (Muri et al., 1991; Wessel and Kompf, 1991), to disturb central fixation (Zangemeister et al., 1995) or to modify saccade or smooth pursuit movement in flight (Wessel and Kompf, 1991). Only under facilitating conditions, e.g., during the performance of a double-step saccade task, has rTMS been reported to be able to induce multistep short-latency eye movements in a few subjects (Li et al., 1997). This result strongly suggests that the organization of the systems within the FEF devoted to eye movement is different than that characterizing the primary motor cortex for limb movements. Indeed, the latter projects directly to spinal motor neurons and can thus easily trigger hand movements when the primary motor cortex is stimulated with TMS. In contrast, circuits leading to gaze movements include intermediate synaptic chains and structures and hence might not be that easy to activate with the same technique. Additionally, it has been also argued that such differences in activation could also be attributed to the fact that the TMS-induced currents may have been either insufficiently high or too poorly focalized to effectively activate polysynaptic chains down to saccadic motor neurons (Muri et al., 1991; Wessel and Kompf, 1991).

Although TMS cannot directly induce eye movements in healthy humans, it can effectively interfere with the processing of visually and non-visually guided saccades. Such modulatory phenomena have been employed to design new causal methods to localize the FEF in healthy humans. In such procedures, a TMS coil is moved around the approximate location of the FEF. Pulses are delivered with intensity generally at or slightly above the resting motor threshold (RMT), i.e., the intensity at which they induce overt evoked hand muscle activations in half of the trials when stimulating the primary motor cortex. Like in microstimulation studies, the choice of intensity is somehow arbitrary. Indeed, it is likely that simulating at 100 or 120% of RMT will lead to different results. More importantly, stimulating at an intensity based on the RMT does not warrant consistent results across participants as it is known that, except under certain circumstances (Deblieck et al., 2008) the TMS-measured excitability of one area is poorly predicting the TMS-measured excitability of another area (Stewart et al., 2001; Boroojerdi et al., 2002; Antal et al., 2004; Kahkonen et al., 2005). Notwithstanding this limitation, TMS procedures allow identifying the FEF as the area in which stimulation significantly modifies some saccadic outcome parameters, generally the latency of a specific type of saccade.

Using such methods, the greatest delays in saccade latencies have been obtained when targeting an area on or 2 cm anterior to the inter-aural line, approximately 6 cm lateral to the vertex, situated between areas over which TMS could generate motor-evoked potentials in hand's and face's muscles (Thickbroom et al., 1996). The authors of these reports did not exclude that the FEF could also extend more rostrally, and that such projections cannot be easily assessed either because rostral stimulation would cause blinks, or because the anterior portions of FEF are involved in other aspects of saccade programming. Other studies localized the FEF within areas situated 2 cm (Ro et al., 1999) or 1.5 cm (Ro et al., 2002) rostral to the motor hand area. However, such site, probably belonging to the middle-frontal gyrus and close to the pre-central sulcus, could not be localized in every tested participant. Moreover, this localization suffers from important interindividual differences, mostly within the coronal or dorsal to medial plane, consistent with reports from neuroimaging studies (Paus, 1996). Studies by O'Shea et al. (2004) and Silvanto et al. (2006) targeting the FEF based on anatomical landmarks within the middle frontal gyrus, just rostral from the junction of the pre-central and the superior frontal sulci, reported that such area corresponds to about 3–4 cm rostral to the individual motor hand area representation. In spite of its rostral location, the reported mean Talairach coordinates locate very close to the coordinates reported by Paus (1996) in their meta-analysis (Table 2). Further work to causally define the FEF location in individual participants by means of TMS employing individualized MRI guidance and studies directly comparing TMS and fMRI FEF localizers within the same population of subjects remain to be performed.

### Consequences for studying motor, visual or cognitive properties of the FEF

Table 1 summarizes the findings for the above-cited literature concerning the search for FEF localization. Variability across species, methods, hemispheres, and individuals in the number of foci associated with FEF and their exact localization raises concerns about how we can explore its role in eye movements or cognitive function.

In TMS studies exploring the causal contributions of FEF in eye movements or cognitive processes such as attentional orienting, consciousness or decision making (see Section on the Role of the FEF), the gold standard would be to use a similar mapping methodology to identify the exact location of this region prior to its manipulation. Based on this notion, for instance, Olk et al. (2006) took the time to identify an area around its *a priori* anatomical location on which TMS induced longer latencies for contralateral than ipsilateral saccades. However, in order to limit the duration of the experiments, other studies employed relative coordinates leading to the average location, expressed as the distance in cm from the motor hand area (which can be easily identified with TMS) and successfully reported significant effects on quantitative measures of eye movements (Wipfli et al., 2001; Nyffeler et al., 2006a,b; Nagel et al., 2008; van Donkelaar et al., 2009). Similarly, in another study, the FEF was localized by probing a series of frontal cortical sites rostral to the motor hand area until evoked hand motor responses disappeared (Leff et al., 2001). Nevertheless, one of the most commonly used strategies consisted in targeting those locations identified in anatomical MRIs by means of on sulci/gyri configurations (O'Shea et al., 2004), or on the basis of normalized coordinates from neuroimaging studies or meta-analyses (Grosbras and Paus, 2002), or employing individual functional localizers based on fMRI acquisitions performed during eye movements tasks (Gagnon et al., 2006).

In conclusion, potential conflicting results across studies concerning the function of the FEF might be related, among other factors, to variability in the way it is localized. This observation has to be kept in mind when interpreting the results that will be presented in the following part.

## Role of FEF

This section will review the role of the FEF in eye movements and in visuo-spatial attention, visual awareness, and perceptual modulation.

### Role of FEF in oculomotor tasks

In humans, knowledge on the role of FEF in several types of eye movements (summarized in Table 3) has been mainly derived from clinical cases in which the FEF has been damaged or from applying TMS on the FEF of healthy persons. These studies are reported in Tables 4, 5 and the conclusions derived from them are reported below.

**Table 3:** **Types of eye movements and experimental paradigms to elicit them**.

**Type of eye movement**	**Paradigm**	**Description**
Spontaneous	In the dark	Movement not triggered toward a visual target
Reflexive (triggered by the sudden appearance of a visual target in space)	Simultaneous	The fixation point switches off and the target appears simultaneously
	Gap (facilitates the most reflexive saccades)	The fixation point switches off and the target appears after a gap period typically lasting a few hundred milliseconds. Such gap period is believed to facilitate fixation disengagement and movement preparation. Typically leads to the gap effect (shorter latency in the gap than the simultaneous paradigm) and express saccades (with latency < 120 ms in humans)
	Overlap	The fixation point remains on the screen after the target appears, for an overlap period in which the two are simultaneously present for a few hundred milliseconds. In such paradigm, there is an enhanced competition between maintaining fixation and preparing a saccade. Typically leads to the overlap effect (i.e., longer latency in the overlap than in the simultaneous paradigm)
	Flashed	The movement is triggered by briefly flashed visual targets toward the location in which they had appeared
Voluntary (the target was already present, is already gone, or was never present)	Visually-guided voluntary	Typically triggered by endogenous cue (such as an auditory signal or a central arrow prompting a saccade toward a lateral target)
	Memory-guided	Participants are required to make an eye movement when a fixation point extinguishes (go signal) toward a target that was flashed before
	Anti-saccade	Participants are required to perform a saccade away from a visual target, which involves the inhibition of a reflexive pro-saccade and the generation of a voluntary, non-visually-guided anti-saccade
Predictive	Repetitions allowing the participant to predict the direction, amplitude and timing of the next target	Movement triggered toward a stimulus not present yet (i.e., with latency < 80 ms in humans)

**Table 4:** **Effects of FEF lesions on eye movements**.

**Type of eye movement**	**Paradigm**	**Effects of FEF lesions**	**Studies**	**Interpretation**
Reflexive saccades	Gap	Shorter latencies	Pierrot-Deseilligny et al., 1987	Disinhibition of the SC
		Longer latencies for ipsilesional saccades	Henik et al., 1994	Disinhibition of ipsilesional midbrain structures and inhibition of contralesional SC
		Normal latencies	Pierrot-Deseilligny et al., 1991; Rivaud et al., 1994; Gaymard et al., 1999	Mild involvement of the FEF in the triggering of the most reflexive saccades
	Briefly flashed targets	Normal latencies	Guitton et al., 1985	
	Overlap	Increased latencies for contralesional saccades	Gaymard et al., 1999	Involvement of the FEF in fixation disengagement and/or the general triggering of pro-saccades
		Increased latencies for ipsilesional saccades	Machado and Rafal, 2004a	
		Increased latencies for both contra- and ipsilesional saccades	Rivaud et al., 1994	
Voluntary saccades	Saccades in response to an arrow cue presented centrally	Increased latencies for contralesional saccades	Henik et al., 1994	Major role of the FEF in triggering voluntary contralateral saccades
	Memory-guided saccades	Increased latencies for bilateral saccades	Rivaud et al., 1994; Gaymard et al., 1999	Involvement of the FEF in fixation disengagement and/or triggering of saccades
Anti-saccades	Anti-saccades	Increased percentage of erroneous pro-saccades toward a contralesioal visual target	Guitton et al., 1985; Machado and Rafal, 2004b	FEF lesions would not only result in a contralesional inhibition of the SC but also in a hypersensitivity of the ipsilesional SC to trigger contralesional saccades
		No enhancement of the percentage of erroneous pro-saccades	Rivaud et al., 1994; Gaymard et al., 1999	Controversial role of the FEF in reflexive saccade inhibition
		Increased latencies for bilateral correct anti-saccades	Rivaud et al., 1994; Gaymard et al., 1999	Involvement of the FEF in triggering voluntary saccades
Predictive saccades	Predictable direction, amplitude and timing	Decreased percentage of contralesional predictive saccades	Rivaud et al., 1994	Importance of the FEF (together with the DLPFC and other subcortical structures) for predictive movements
Other eye movement parameters (gain)	Predictive, memory-guided & reflexive saccades	Deteriorated gain of contralesional saccades	Rivaud et al., 1994; Gaymard et al., 1999	Involvement of the FEF in the computation of retinotopic saccades (for which the target location is determined in respect to the position of the eye, see Pierrot-Deseilligny et al., 1995)
	Smooth pursuit, OKN	Deteriorated gain of ipsilesional smooth pursuit and OKN	Rivaud et al., 1994	Involvement of the FEF in the computation of other types of eye movements

**Table 5:** **Effects of TMS over the FEF on eye movements**.

**Type of saccades**	**TMS delivery**	**Effects of FEF stimulation**	**Studies**	**Interpretation**
Reflexive saccades	60–100 ms after target onset	No effect on latencies	Muri et al., 1991	
	60 ms before expected movement	Longer latencies (but preserved express saccades)	Priori et al., 1993	Circular coil centered over the vertex probably influencing several cortical areas among which the FEF, SEF and PPC
	Middle or end of a 200-ms gap interval	Longer latencies (mainly of contralateral saccades)	Nagel et al., 2008	Interference with motor preparation during the gap period (also when stimulating SEF and DLPFC; cortico-cortical or cortico-subcortical networks)
	50 ms period around target onset	Shorter latencies of ipsilateral saccades (but at the expense of precision; multiple saccades)	van Donkelaar et al., 2009	FEF (and left SEF) preventing the release of a saccade until its planning has been completed
Reflexive saccades (with a voluntary component)	From target onset to 100 ms after	Shorter/longer latencies of contralateral/bilateral saccades depending on TMS timing and paradigm	Nyffeler et al., 2004	Facilitatory effects: suppression of fixation activity (within the SC). Disruptive effects: interference with the burst saccadic signal
	rTMS to decrease cortical excitability	Longer latencies of bilateral saccades	Nyffeler et al., 2006a,b	Impairment of fixation disengagement and of burst signal (in the stimulated FEF and/or the contralateral FEF)
	rTMS to decrease cortical excitability	Shorter latencies of bilateral saccades	Gerits et al. (2011) in monkeys but see Pouget et al. (2011)	Suppression of fixation neurons in the FEF; rTMS might impact both FEF via transcallosal connection
Voluntary saccades	50 ms before expected movement	Longer latencies of contralateral saccades	Thickbroom et al., 1996	Interference with programming and execution of saccades
	from 100 before to 100 ms after go signal	Longer latencies of contralateral saccades	Ro et al., 1997, 1999, 2002	Interference with the programming and the execution of saccades (including perceptual analysis of the go signal)
Anti-saccades	50–90 ms after target onset	Longer latencies of ipsilateral anti-saccades (bilateral in females)	Muri et al., 1991	Reduced attention in the contralateral visual field or insufficient suppression of reflexive saccades
	100 ms after go signal	Longer latencies of bilateral anti-saccades (and enhancement of erroneous contralateral pro-saccades)	Terao et al., 1998	Interference with the emergence of the motor signal (interhemispheric transfer of information)
	Between 50 and 150 ms after target onset	Longer latencies of ipsilateral anti-saccades	Olk et al., 2006	Interference with saccade inhibition to the contralateral visual field
	Middle or end of a 200-ms gap interval	Longer latencies (mainly of contralateral saccades)	Nagel et al., 2008	Interference with motor preparation during the gap period (also when stimulating SEF and DLPFC)
	150 ms after target onset	Shorter latencies (sometimes longer latencies, depending on animals, TMS intensity and saccade direction)	Valero-Cabre et al. (2012), in monkeys	Modulatory (likely suppressive) effect of FEF fixation neurons
Memory-guided movements	At go signal and 50 ms later (double-pulse)	Shorter latencies of contralateral saccades	Wipfli et al., 2001	Modification of the pre- saccadic build-up activity or inhibition of suppression cells in the FEF
	100 ms after go signal	Longer latencies of memory-guided saccades, vergence and both components of combined saccade-vergence movements	Yang and Kapoula, 2011	Interference with fixation disengagement or with premotor memory activity. FEF involved in all rapid eye movements in 3D space
Other eye movement parameters	Various	No effect of TMS on saccade precision or velocity	Most of studies (e.g., Priori et al., 1993)	
	From 100 to 50 ms before saccade onset	Suppression of saccades or longer latencies associated with increased duration and smaller velocity	Zangemeister et al., 1995	Shortening of the saccadic burst (clear effect after TMS at multiple locations but larger when stimulating parieto-occipital regions)
	50 ms period around target onset	Multiple small short-latency ipsilateral saccades instead a unique large one	van Donkelaar et al., 2009	FEF (and left SEF) preventing the release of a saccade until its planning has been completed
	At various timings	Smaller or higher gain (velocity) of a sinusoidal predictive pursuit depending on TMS timing	Gagnon et al., 2006	FEF also contributing to the computation of eye movements dynamics
	rTMS to decrease cortical excitability	Smaller gain of ipsilateral memory-guided anti-saccade	Jaun-Frutiger et al., 2013	FEF participating in visual vector inversion during the anti-saccade task

#### Lesions studies

Most of lesion studies describing the role of the FEF have been focusing on oculomotor deficits that are reported in Table 4. The general pictures emerging from this literature is that FEF lesions very mildly affect the most reflexive saccades but might delay eye movements for which a voluntary component is introduced, for instance concerning fixation disengagement. Thus, although the triggering of reflexive saccades is more likely under the control of the PPC (Pierrot-Deseilligny et al., 2004; Muri and Nyffeler, 2008), the FEF could still play a role, revealed under specific cognitive conditions. In that vein, the FEF has been hypothesized to play a context-dependent modulatory influence over different cortical and subcortical structures involved in different categories of reflexive saccades. Such role could be revealed by switching cost or benefit when alternating between gap and overlap pro-saccades (Vernet et al., 2009). The role of FEF in reflexive saccade inhibition remains controversial, the DLPFC being a more likely candidate to control such inhibition (Pierrot-Deseilligny et al., 2004; Muri and Nyffeler, 2008). Finally, the FEF (together with the DLPFC and other subcortical structures) is more commonly thought as a controller for voluntary saccades such as predictive, memory-guided and anti-saccades (Pierrot-Deseilligny et al., 2004; Muri and Nyffeler, 2008). In addition, the FEF is involved in the computation of the amplitude of all types of eye movements.

Despite their undeniable value, several aspects limit the strength of the conclusions that can be drawn from lesion studies. First, lesions are rarely limited to the FEF, making it difficult to isolate the specific involvement of the FEF in the observed deficits. Second, different deficits might be observed during the acute and chronic phase following the lesions. Transient hypo-perfusion of areas connected to the damaged area, a phenomenon known as diaschisis or, on the contrary, complex plastic reorganization within the impaired network, render the role of the FEF difficult to isolate from the role of the entire network. Other cortical and subcortical areas, or the contralesional FEF, seem to play an important role in developing compensatory mechanisms (for a review see Muri and Nyffeler, 2008). In monkey studies, in which more spatially precise transient inactivation or lesions can be performed, acutely observed deficits (Sommer and Tehovnik, 1997; Dias and Segraves, 1999) rapidly disappeared, except for complex tasks such as memory-guided saccades or saccades toward flashed targets, or if lesions to the FEF were combined with lesions to other areas (for a review see Tehovnik et al., 2000; Muri and Nyffeler, 2008).

#### TMS studies

The most commonly reported effect of TMS over the FEF during a saccadic task is a modulation of its preparation latency. Because of the alerting effect linked to the clicking sound and taping sensation associated with the coil discharge, it is known that TMS can have unspecific (i.e., not related to the effects of the electrical currents induced on brain tissue) effects on reaction times and eye movement latencies. Thus, shorter latencies could be related to crossmodal facilitation, whereas longer latencies could result from the participants waiting for TMS discharge as for a “go” signal. Thus, it is important to ensure that the effects on latencies are either stronger or in the reverse direction than the effects obtained in a control condition, such as sham stimulation or the active stimulation of a control brain area unrelated to saccadic control or execution. Using such cautionary measures, TMS over FEF has been shown to modulate the latency of different types of saccades.

TMS studies exploring the role of FEF in eye movements are reported in Table 5. As with patients' studies, whether TMS over FEF can delay reflexive saccades toward suddenly appearing visual targets remains unclear and most of the effects on latency modulations have been shown on pro-saccades involving some degree of voluntary or intentional component. In anti-saccade modulations, whether TMS stimulation of the FEF disturbs the suppression of the reflexive pro-saccade or the preparation of the voluntary anti-saccade or both is not entirely clear. In general, TMS is believed to interfere with several stages in the execution of saccades, including the perceptual analysis of the cues or targets and the motor preparation (burst signal). Occasional facilitatory effects on saccade latency have been attributed to suppression of fixation activity (within the FEF or the FEF projections to the SC). While most reports demonstrated, in accordance with microstimulation studies, effects on contralateral saccades, some studies demonstrate ipsilateral or bilateral effects that could be related to a modulation of fixation cells activity or to transcallosal modulation of both FEFs. Interestingly, TMS can modulate the latency of several eye movements performed in 3D space. Finally, the FEF is not only involved in fixation, fixation release and the triggering of voluntary eye movements but also contributes to the computation of eye movements dynamics (gain, velocity).

In conclusion, non-invasive neurostimulation studies employing TMS largely confirmed, in healthy humans and with higher spatial and temporal resolution, the insights drawn from patient studies. The unquestioned role of the FEF in the triggering of voluntary eye movements as well as the still controversial role of this region in reflexive movement inhibition and initiation is reminiscent of the blurred frontiers between reflexive and voluntary movements and of the importance of entire oculomotor networks for the control of eye movements, in which the relative contribution of each node is modulated by the cognitive context. The rest of this Section on the Role of FEF will explore how the FEF is involved in a very diverse set of higher cognitive functions (see also Table 6 reporting TMS studies on these topics).

**Table 6:** **Effects of TMS over the FEF on visuo-spatial attention, visual awareness and perceptual modulation**.

**Function /Task**	**TMS parameters**	**Effects**	**Studies**	**Interpretation**
Saliency map	Single-pulses over the right FEF	Increased the distractor-related deviation of saccade trajectory	Walker et al., 2009	FEF participates in the elaboration of a saliency map (enhancement of target-related activity and/or suppression of distractor-related activity)
Coupling between attention and eye movements	2 pulses separated by 40 ms over the right FEF	Delayed saccade latency for TMS applied in two time windows (early for pro-saccades and late for pro- and anti-saccade)	Juan et al., 2008	Distinct visual stimulus processing (early time window) and saccade preparation (late time window), hence dissociation between attention and motor preparation
	3 pulses at 33 Hz or single-pulses over the right or left FEF	Modulation of discrimination performance on locations to which eye movements are being prepared (when contralateral to TMS location)	Neggers et al., 2007; Van Ettinger-Veenstra et al., 2009	The coupling between attention and eye movements can be modulated by TMS
Visual search, spatial priming (and working memory), switch detection	TMS at 10 Hz for 500 ms or double-pulses over the right FEF	Disrupted visual search	Muggleton et al., 2003; O'Shea et al., 2004; Kalla et al., 2008	Right FEF is involved in visual search, particularly when the visual target is neither salient nor predictable.
	TMS at 10 Hz for 500 ms over the left FEF	Disrupted spatial priming; increased color switch costs	O'Shea et al., 2007; Campana et al., 2007; Muggleton et al., 2010	Left FEF would be an area of convergence and integration of memory traces during the preparation of an overt motor response
	TMS at 10 Hz for 500 ms over the right or left FEF	Disrupted spatial priming by right (but not left) TMS and disrupted visual search by right (but not left) FEF TMS stimulation and disrupted. Effects found for both near and far space	Lane et al., 2012, 2013	Right and left FEF involved in visual search; right FEF involved in spatial priming. Effects are depth-independent (near/far space)
	single-pulses over the right or left FEF	disrupted trans-saccadic memory of multiple objects	Prime et al., 2010	FEF is involved in spatial working memory (or there is a spatial working memory area near the FEF)
Top-down influence of the FEF on visual areas	5 pulses at 10 Hz over the right FEF (TMS-EEG experiment)	modulated attention-related ongoing EEG activity as well as visual-evoked pontentials	Taylor et al., 2007	FEF modulates the excitability of visual areas
	5 pulses at 9 Hz over the right FEF (TMS-fMRI experiment)	modulated BOLD activity within areas V1-V4 (increased for peripheral and decreased for central visual field); enhanced perceived contrast for peripheral relative to central visual stimuli	Ruff et al., 2006	
	1 conditioning pulse over the right or left FEF 20–40 ms before 1 test pulse over MT/V5	Conditioning pulse decreased the intensity needed for the test pulse to induce a phosphene	Silvanto et al., 2006	
Visual discrimination, detection, awareness	Single-pulses over the right or left FEF	Decreased RT or increased sensitivity, bilaterally (i.e., for right and left targets) after right FEF stimulation and contralaterally (i.e., for left targets) after left FEF stimulation. Effects modulated by attention and shaped by individual connectivity	Grosbras and Paus, 2002, 2003; Chanes et al., 2012; Quentin et al., 2013	TMS over the FEF increases background activity (brings it closer to a perceptual threshold) and/or boosts relevant neural population
	4 pulses at 30 Hz or 50 Hz over the right FEF	30 Hz stimulation increased sensitivity; 50 Hz stimulation relaxed response criterion. Effects shaped by individual differences of fronto-parietal connectivity between the FEF and the IPS	Chanes et al., 2013; Quentin et al., 2014	There is a frequency multiplexing of several functions within the FEF; TMS entraining rhythmic activity potentially mimicks attentional effects
	5 pulses at 20 Hz over the left FEF	Decreased the RT cost of invalid cueing before contralateral target	Smith et al., 2005	As TMS increased performance at cued locations, it also disrupts inhibition of processing at unattended location

### Visual activity and saliency map within the FEF

The FEF encodes visual signals and is believed to participate in the visuo-motor transformation for the preparation of eye movements, as suggested by the influence of FEF on the accuracy of eye movements (see effects of lesions and TMS on other eye movement's parameters in Tables 4, 5). Beyond this contribution, the FEF can be considered as a visual area in itself, with early visual-evoked responses reported in anesthetized animals, peaking even before activity reaches V2 or V4 (Schmolesky et al., 1998). Moreover, the projections from the FEF to V4 could be characterized as feed-forward connections, i.e., going from lower to higher hierarchical levels (Barone et al., 2000). Wurtz and Mohler (1976) reported that some of the visual cells within the FEF displayed an enhanced response to a visual stimulus when a saccade was made toward the receptive field rather than away from it. Such selective enhancement would demonstrate the ability of the FEF (and similarly, also that of the SC) to evaluate stimulus significance and use such information for saccade preparation. Although there is a clear relationship between visual and movement properties of the FEF in terms of spatial selection, there is also some degree of dissociation. Bruce and Goldberg (1985) described in the FEF a continuum of visuo-motor cells, from purely visual to purely motor cells, the latter cells being less sharply tuned to direction and amplitude than the former cells, and with visuo-motor cells showing intermediate tuning. In humans, Blanke et al. (1999), recording with intracranial electrodes visual-evoked potentials in epileptic patients, showed strong visual responses for contralateral visual stimuli (consistent with the direction of the electrically-elicited eye movements) but also responses of lower amplitude after ipsilateral visual stimulation.

The visual activity encoded within the FEF has been primarily related to the computing of a saliency map, where neural activity codes for the location of a behaviorally relevant target displayed among distractors during a typical visual search task (for a review see Schall and Bichot, 1998; Thompson and Bichot, 2005). There would be a gradual suppression of distractor-related activity paralleled by an enhancement of target-related activity. Saccades are generally performed toward the “winner” of this saliency map. However, similar computations are performed even when no saccades are required or when a saccade should be performed away from the ultimately selected “winner” target. Indeed, in a go/no go visual search task, although visual response within the FEF is enhanced when the saccade is executed (go trials), discrimination of the target occurs within similar timing in both go and no go trials (Thompson et al., 1997). There would be an early (around 50 ms) non-discriminative visual response within the FEF followed by a later (around 100–150 ms) discriminative selection of the target among distractor regardless of its visual features (Thompson et al., 1996; Thompson and Bichot, 2005), but even the early response can show discriminative properties in experienced animals (Bichot et al., 1996). When saccades are triggered toward the target, the variability in saccade latencies is poorly correlated with the speed of discrimination of the FEF cells and seems to be rather related to distinct motor preparation stages (Thompson et al., 1996).

Walker et al. (2009) brought direct causal evidence in humans that the FEF might be participating in the elaboration of a saliency map for the selection of a target of an upcoming saccade. Indeed, when a competing visual distractor appeared in the same direction as the saccade goal but at unpredictable locations, saccade trajectories deviated away from the distractor. The magnitude of this distractor-related deviation of saccade trajectory was increased by single-pulse TMS over the right FEF. The interpretation is that stimulation of the FEF might have disrupted the process of enhancing target salience or could have increased the inhibition associated with the distractor.

### The FEF at the heart of the coupling between attention and eye movements?

The saliency map described above could reflect the deployment of visuo-spatial attention. The premotor theory of attention postulates that orientation of spatial attention derives from the same mechanisms dedicated to action: attention is oriented to a given point in space when the oculomotor program for moving the eyes to that point is ready to be executed (Rizzolatti et al., 1987). In this perspective, FEF “visual” activation could be attributed to the preparation of saccade programs, which may or may not be overtly executed, rather than to the visual analytic processes in the FEF.

Many behavioral and neurophysiological studies support this theory, according to which covert attention shifts without eye movements, conceived as a specific and distinct process with a mechanism of its own, might simply be an artificial separation of otherwise unified underlying processes. Among the many behavioral pieces of evidences in accordance with the premotor theory of attention, one could cite the enhanced visual discrimination performance when a discrimination stimulus and a saccade target converge to the same object whereas it declines steeply when they refer to items at different locations, arguing against the ability to direct visual attention to one location while simultaneously preparing a saccade towards another location (Deubel and Schneider, 1996). Neuroimaging studies often find similar activations, including in the FEF, for eye movements and attentional shifts, and a remarkable level of overlap of the underlying circuits of these operations, as summarized in a meta-analysis on PET and fMRI studies (Grosbras et al., 2005). Interestingly, the involvement of the FEF in contralateral attention shifts would be particularly marked when participants have to overtly respond to a target, for instance with a manual response (Corbetta et al., 1993) or when the attentional task is particularly demanding (Donner et al., 2000).

However, there is also evidence against a strict interpretation of the premotor theory of attention. For instance, TMS during saccade preparation was able to modulate discrimination performance at the target location: while TMS over the intraparietal sulcus (IPS) ipsilateral to the saccade's direction increased general performance, non-invasive stimulation over the FEF contralateral to the saccade's direction specifically decreased or enhanced discrimination on the target location depending on the exact stimulation parameters (Neggers et al., 2007; Van Ettinger-Veenstra et al., 2009). Thus, the FEF plays a role in mediating the coupling between visuo-spatial attention and eye movements and such coupling can be modulated by TMS (Neggers et al., 2007; Van Ettinger-Veenstra et al., 2009). Other arguments against a motor preparation toward the target location to which attention is oriented can be found in microstimulation experiments with monkeys (Juan et al., 2004) or TMS experiments in humans (Juan et al., 2008). In the first study (Juan et al., 2004), monkeys had to perform a visual search and a saccade toward (pro-saccade) or away from (anti-saccade) a visual target depending on its orientation. Microstimulation of the FEF at variable timings after target onset evoked, in anti-saccade trials, saccades progressively toward the endpoint of the correct saccades but never toward the visual target. Using a similar task in humans, Juan et al. (2008) showed that double-pulse TMS over the right FEF can delay saccade latencies in two distinct time-windows: an early window (40–80 ms after target onset) in which the delay in pro-saccades was interpreted as a disruption of the visual stimulus processing and also a late window (200–160 ms before the expected saccades) in which a delay in pro- and anti-saccades was interpreted as a disruption of saccade preparation.

### Context-dependent role of FEF during visual search

Whether or not eye movement preparation is strictly linked to attention orientation does not question the involvement of the FEF in visual discrimination performance, either directly or indirectly though its massive set of anatomical projections toward the visual cortex. Several TMS studies in humans have been designed to accurately describe the role of the left and right FEF in covert voluntary attentional orienting and visual discrimination performance. For instance, Muggleton et al. (2003) showed that rTMS at 10 Hz for 500 ms over the right FEF during the presentation of a search array disrupted visual search. These authors showed that a decrease of the visual sensitivity explained by a higher number of false positives (i.e., incorrect detections reported by participants when the target was absent) and attributed to a reduced ability to process the items. Interestingly, only specific subtypes of visual search impaired by the stimulation, such as conjunction search was impaired (i.e., when the target shares the same color than about half of the distractors and the same orientation than the remaining distractors) and, to a lesser extent, interleaved feature search (i.e., when the color of target and distractors is randomly attributed at each trial). On the contrary, rTMS had no effect on constant feature search (i.e., when the target and distractors always look the same across trials). The authors concluded that the right FEF is particularly important for visual search when the visual target is neither salient nor predictable. Using double-pulse TMS paradigms, such findings were confirmed for an early time window of up to 80 ms after search array onset, i.e., much earlier than the involvement of the PPC in visual search (O'Shea et al., 2004; Kalla et al., 2008).

Using similar visual search paradigms, the role of FEF in visual priming (form of implicit memory that facilitates the detection of a target that shares common features with a recently inspected search target) or, on the contrary in switch detection has also been addressed in TMS approaches. Indeed, fMRI experiments reported a suppression of BOLD response in fronto-parietal networks, including the FEF, during simultaneous color and location repetition (Kristjansson et al., 2007). Non-invasive brain stimulation studies showed that 10 Hz rTMS patterns for 500 ms over the left (but not the right) FEF disrupted spatial priming, as measured by increased reaction times, when applied during the presentation of the search array (O'Shea et al., 2007) or during the inter-trial interval (Campana et al., 2007). This result suggested that the memory trace is probably distributed through visual and oculomotor networks typically required for those behaviors and that the FEF would be an area of convergence and integration during the preparation of an overt response (O'Shea et al., 2007). Finally, the left FEF would also be involved in the ability to detect a color switch (or select a new target) as identical rTMS patterns delivered to the left FEF applied in-between trials increased switching costs by slowing down the response time for switch trials (Muggleton et al., 2010).

However, the right/left hemisphere frontal asymmetries described above are questioned by other studies showing that rTMS at 10 Hz for 500 ms, over the right but not the left FEF, from the beginning of an array onset, disrupts spatial priming and that similar rTMS over both right and left FEF increases reaction time when the target position is random (Lane et al., 2012). Interestingly, the same team also demonstrated that such involvement of the right FEF is independent of the depth (near vs. far space) at which the task is performed, whereas the right PPC would be involved in near space and right ventral occipital cortex in far space (Lane et al., 2013).

Finally, it is possible that the FEF is more directly involved in spatial memory, in particular in trans-saccadic memory. Indeed, Prime et al. (2010) showed a decrease of the number of items participants could remember when left or right FEF were disrupted around saccadic time. Such effect could however be related to the stimulation of a spatial working memory area that has been identified just rostral to the FEF (Courtney et al., 1998).

### Top-down modulation of visual areas

Several studies suggested that the contributions of the FEF to discrimination performance are mediated by its output projections to the visual cortex. Indeed, electrophysiological evidence in both animals and humans demonstrated a relation between activity within the FEF and excitability of occipital brain areas. Moore and Armstrong (2003) showed that the intracortical stimulation of the FEF in monkeys at current intensities below those required to evoke saccades (i.e. subthreshold stimulation), enhanced visual responses in visual area V4. Such enhancements were retinotopically specific. If the endpoint of the saccade evoked by suprathreshold stimulation of the FEF overlapped with the receptive field of a V4 cell, subthreshold FEF stimulation enhanced this V4 cell's visual responses. This type of top-down modulation of visual cortex excitability could explain earlier findings in non-human primates consisting in enhanced perception (decreased threshold for detecting a luminance change) of peripheral visual stimulus after subthreshold FEF stimulation, only when the visual stimulus was displayed within the “motor field” of the stimulated FEF (Moore and Fallah, 2001).

Although TMS cannot reach the spatial resolution required to target neural populations within the FEF subtending specific visual or motor fields, several studies in healthy humans, combining TMS with EEG (Taylor et al., 2007), TMS with fMRI (Ruff et al., 2006) or employing double coil TMS and psychophysics, showed similar top-down influence of the FEF on visual areas and visual performance. Short 5-pulse trains of 10 Hz rTMS applied over the right FEF during a cueing period of a covert orienting task modulated attention-related ongoing EEG posterior potentials before visual stimulation, as well as the potentials evoked by the visual stimulus (Taylor et al., 2007). Similar short 5-pulse trains of 9 Hz TMS over the right FEF modulated the BOLD activity recorded with fMRI within visual areas V1-V4 led to activity increases for retinotopic representations of the peripheral visual fields combined with activity decreases of central retinotopic locations (Ruff et al., 2006). A follow up experiment showed that TMS over the right FEF enhanced perceived contrast for peripheral relative to central visual stimuli (Ruff et al., 2006), hence proving that such activity modulation was behaviorally relevant. Finally, the double-coil TMS technique can be used to simultaneously induce activity within the left or right FEF and measure the excitability of MT/V5. Stimulation of the FEF 20–40 ms prior to stimulation of MT/V5 decreased the intensity of MT/V5 stimulation required to elicit phosphenes, demonstrating that the FEF has a direct modulatory effect on the excitability of this motion visual area (Silvanto et al., 2006).

### Modulation of visual performance and awareness

In line with animal and human studies showing respectively, enhanced perception and increased activity in visual areas following FEF stimulation, several reports have also shown that TMS over the left and right FEF was able to speed-up discrimination and/or increase detection and visual awareness (Grosbras and Paus, 2002, 2003; Chanes et al., 2012). Grosbras and Paus (2002) reported that a TMS pulse delivered to the left or right FEF 53 ms prior to target onset could decrease reaction time in a forced-choice discrimination task. These same authors showed that a TMS pulse delivered to the left or right FEF 40 ms before the onset of a masked target could also increase sensitivity in a visual detection task (Grosbras and Paus, 2003). Similarly, Chanes et al. (2012) provided evidence showing that a TMS pulse delivered to the right FEF 80 ms before a low-contrast target could increase visual perceptual sensitivity in a detection task. These studies have shown that right FEF stimulation generally leads to bilateral effects whereas left FEF stimulation results in an increase of performance solely for stimuli presented in the contralateral visual hemifield (Grosbras and Paus, 2002, 2003; Chanes et al., 2012). In addition, interactions between TMS effects and the manipulation of visuo-spatial attentional orienting in space before target presentation have been found in studies that combined a strategy to modulate attentional processes by means of spatially informative visual cues and by means of non-invasive neurostimulation delivered to specific cortical regions. More specifically, the increase of performance after right FEF stimulation reported in the above-mentioned studies occurred specifically for validly cued (or attended) locations, and also following spatially neutral cues, but not for unattended locations following invalid cueing (Grosbras and Paus, 2002; Chanes et al., 2012).

Enhancement of perception may result from a global increase of background activity, drifting closer to a perceptual threshold, hence allowing any incoming weak signal to reach it more easily; in addition to this global injection of activity, TMS outcomes are also highly dependent on the state of the targeted regions and their mixed populations of neurons. Accordingly, TMS may selectively boost specific clusters of neurons according to their level of activity (O'Shea and Walsh, 2004). In this context, prior reports have suggested that the visual performance and awareness enhancement occurred directly by changing activity in FEF (manipulating genuine processes purported by this frontal region) or indirectly *via* connections between the FEF and visual regions modulating the input gain of incoming visual signals (Grosbras and Paus, 2003). Interestingly, interindividual differences in the direction and magnitude of the TMS driven facilitatory effects reported in Chanes et al. (2012) correlated significantly with the probability of anatomical connection between the FEF and the SC estimated by means of white matter probabilistic tractography. Such result suggests a key role for white matter connectivity between the stimulated area and other key brain structures to explain at the network level the strength of TMS modulatory influences on visual performance (Quentin et al., 2013).

It was also suggested that TMS effects could actually enhance perception in a way similar to what occurs naturally during cover shifts of attention (Grosbras and Paus, 2003). Following that line of thought, Chanes et al. (2013) conceived an experiment in which TMS was used as a way to emulate activity that would mimic neurophysiological spatio-temporal patterns signaling processes of attentional orienting. This study was based on a prior report by Buschman and Miller (2007) demonstrating, in non-human primates, high-beta (~30 Hz) and gamma (~50 Hz) fronto-parietal synchronizations subtending top-down and bottom-up attentional processes, respectively. By using short trains of stimulation at those same frequencies, Chanes et al. (2013) observed an increase of perceptual sensitivity during a low-contrast target detection task following right-FEF stimulation at 30 Hz, which would be consistent with an increase of endogenous attention and/or facilitated access to visual consciousness. Interestingly, the strength of the individual TMS improvements correlated significantly with the volume of the first branch of the superior longitudinal fasciculus, which links the stimulated FEF with areas of the posterior parietal cortex within and in the vicinity of the IPS, supporting the idea of a frequency-specific fronto-parietal synchronization induced by rhythmic TMS subtending visual performance ameliorations (Quentin et al., 2014). Moreover, stimulation of the right FEF at 50 Hz induced a relaxation of response criterion, as if sensory evidence in favor of target presence were increased whether or not the target was actually present (Chanes et al., 2013). This result provides support for a frequency-based multiplexing of two distinct processes, such as visual sensitivity and response criterion with bearing on visual performance, emerging from neuronal resources within the same area. Whereas short trains of rTMS have been widely used to drive stronger behavioral effects (see e.g., Smith et al., 2005), they can also be applied, as in Chanes et al. (2013), as a novel way to manipulate rhythmic brain activity, in line with evidence of oscillatory entrainment with such technique (Thut et al., 2011).

To reconcile these studies showing improvements in visual perception and awareness with TMS of the FEF with previously cited reports showing impairment of visual discrimination during visual search tasks (e.g., Muggleton et al., 2003; O'Shea et al., 2004; Kalla et al., 2008), it should be reminded that TMS lacks the spatial resolution to selectively enhance perception in one particular area of the perceptual space. Thus in visual search paradigms, distractors might benefit as much as targets from TMS-driven visual enhancement, decreasing the relative benefit for the latter and leading to perceptual impairments instead of enhancements.

Along the same lines, in addition to increased visual performance at cued locations, TMS over the FEF should disrupt the inhibition of stimulus processing at unattended locations. This hypothesis has been confirmed by the study by Smith et al. (2005), that in agreement with this notion, showed that in a visual detection task, short trains of rTMS at 20 Hz for 200 ms starting 50 ms before cue onset (and not around the timing of target onset as in the previously cited studies) over the left FEF were able to decrease the reaction time cost of invalid cueing before contralateral targets. Such disruption of the inhibition for unattended locations could also explain the results reported by Ro et al. (2003) who showed that single TMS pulses, delivered over the right FEF, showing that single 600 ms after the cue and 150 ms prior to target onset, decreased the inhibition of return phenomenon. This well-known attentional process consists in a worsening of visual performance at locations that had been cued a certain interval of time preceding target onset. It is probably caused by a disengagement of attention and is thought to prevent the re-exploration of an already scrutinized region of the space. The above-mentioned studies support the notion that increased visual detection performance at unattended spatial locations could result from TMS interfering with active mechanisms of inhibition and exploration suppression of unattended spatial locations subtended by the FEF.

To summarize, the FEF cannot be only conceived as an area important for preparing and triggering eye movements but also as an essential region contributing to cognitive processes such as attentional orienting, visual awareness, conscious access, perceptual performance, and decision making. However, as mentioned before, these processes are probably mediated by activity within largely distributed cortico-cortical and cortico-subcortical networks. In particular, fronto-parietal systems are particularly relevant, and effects similar to those of the FEF reviewed in this paper, have been found in specific PPC regions such as the IPS (Chica et al., 2011; Bourgeois et al., 2013a,b). If there is full agreement on the fact that dorsal frontal and posterior parietal areas operate commonly and in synchrony in attentional and visual performance modulation processes, other studies emphasize the differences between the contributions of these two regions. For instance, whereas the dominance of the right PPC in attentional orienting tasks is well known, inter-hemispheric asymmetry is less evident with regards to the contributions of the FEF (Gitelman et al., 1999), and such aspect might prove highly task dependent. Moreover, the FEF could be more involved than the IPS in intentional behavior when overt responses are required (Corbetta et al., 1993). Future studies will be necessary to further understand the common and distinctive role of these two highly interconnected areas.

Finally, it should be mentioned that although the FEF is involved in both eye movements and visual cognition, few studies have explicitly explored simultaneously the combined role of the FEF in both types of function (with the exception of the studies reviewed in a prior section addressing the role of the FEF in the coupling between attention and eye movements). The cognitive context modulates the role of the FEF in eye movements but direct report of conscious perception, for instance, is not performed in eye movements studies. Conversely, studies on cognition rarely explore eye movements (even if correct visual fixation is often assessed with eye-trackers). In future studies, exploration of microsaccades and other fixation eye movements (Martinez-Conde et al., 2013) might shed new light on the relevance of the experimental dissociation between eye movements and cognition.

## Improving visual perception and awareness

In the last part of this review, we will briefly address some links between FEF activity, eye movements and visuo-spatial awareness. Such evidence will allow us to elaborate on the rational behind the potential use of non-invasive brain stimulation and eye movement training in 3D space for the treatment and rehabilitation of visuo-spatial disorders. As an example, we will focus on the case of hemi-spatial visual neglect, often suffered by patients after right hemisphere damage.

### Deficits of visuo-spatial awareness: the case of hemispatial neglect

As reviewed above, the FEF is not only a key node contributing to the planning and execution of eye movements but it is also involved in attentional orienting and several aspects of visual cognition. Surprisingly however, a large majority of the FEF lesion studies in non-human and human primates focused on the consequences of frontal damage on oculomotor deficits, neglecting the exploration of other behavioral consequences. Nevertheless, it has been shown that lesions damaging right attentional networks can often induce attentional orienting and visual awareness disorders such as visuo-spatial neglect. This condition is a highly impairing syndrome consisting in an inability to orient attention to regions of the contralesional space and thus become aware of sensory stimuli presented herein. It is common after stroke lesions impacting cortical or subcortical regions, particularly in the right hemisphere. In a multicenter study developed in a cohort of 1281 acute stroke patients, signs of visuo-spatial neglect occurred in 43% of right brain-injured patients and also in 20% of left brain-damaged patients. Apparent spontaneous recovery of these deficits seems often to occur, but does not necessarily eliminate all signs and deficits, particularly visual extinction, a fact that becomes evident when more challenging and robust-to-learning computer-based tasks are employed instead of paper and pencil tasks to evaluate patient status. Indeed, at 3 months, signs of moderate neglect are still present in 17% of right brain-injured patients and 5% of left brain-injured patients (Ringman et al., 2004). Neglect interferes with the rehabilitation of deficits in other domains, such as motor and sensory, that can also be present in such patients and, if it endures, it can lead to poor clinical recovery outcomes and preclude a reintegration to normal or adapted life. However, despite considerable therapeutic advances in behavioral, sensorial and pharmacological treatments, many patients remain enduringly impaired after rehabilitation (Fierro et al., 2006).

One of the most influential hypotheses to understand visuo-spatial neglect suggests the existence of an impairment in the balance between the orienting attentional bias of each hemisphere towards the contralateral hemispace (Kinsbourne, 1970). This explanation has received support from animal studies (Sprague, 1966; Rizzolatti et al., 1983). In humans, a revealing single patient case revealed how a second stroke involving the left FEF a few days after a right parietal stroke was able to fully compensate the clinical signs of severe neglect induced by the first lesion (Vuilleumier et al., 1996).

### Non-invasive brain stimulation to improve visuo-spatial awareness

In the context of a hemispheric imbalance, the use of non-invasive brain stimulation, namely rTMS and transcranial direct current stimulation (tDCS) has proven particularly promising. Indeed, both techniques have demonstrated efficacy in modulating transiently brain regional excitability (Nitsche et al., 2008; Rossi et al., 2009). Inhibitory stimulation over the intact hemisphere (mostly at the level of parietal areas) has shown promise in decreasing the excessive inhibition over viable regions of the damaged hemisphere and by virtue of this effect relieving neglect symptoms (e.g., Oliveri et al., 2001; Sparing et al., 2009; Cazzoli et al., 2012; Koch et al., 2012).

However, the ability of FEF-TMS to increase visual perception and awareness, at least in healthy subjects on a trial-by-trial basis (Grosbras and Paus, 2002, 2003; Chanes et al., 2012) could revive the interest in targeting this node of the attentional network for rehabilitation purposes. Indeed, neglect is a “network” impairment: there is a striking variability of areas whose lesions results in neglect. Temporal, parietal or frontal cortical lesions or subcortical lesions, or combined lesions of these areas are the most likely to induce enduring neglect 3 months post-stroke (Ringman et al., 2004). Aside from the impairments resulting from the lesions themselves, neglect symptoms could also arise from diaschisis (i.e., abolition of neural activity in areas that are distant but anatomically connected to the lesioned area) and disconnections/hypoperfusion affecting the whole fronto-parietal network. Recent evidence from an fMRI study suggests that functional connectivity in damage and also intact fronto-parietal attentional orienting networks is impaired in neglect patients (He et al., 2007) and the disconnection theory should be strongly considered to explain the pathophysiology of neglect (Doricchi et al., 2008). A recent explanatory model of visuospatial neglect (Corbetta and Shulman, 2011) suggests that neglect primarily arises from damage to the right hemisphere-dominant non-spatial ventral attention network. This ventral network would be interacting with the dorsal fronto-parietal network (encompassing the FEF and the IPS) that controls spatial attention. Thus, structural damage to the right ventral network would result in functional resting and task-related activity asymmetries in the dorsal network, leading to the typical lateralized attention deficit of neglect syndrome.

The existence of fronto-parietal synchronization at specific frequencies associated with attentional processes (Buschman and Miller, 2007) and the possibility to inject such rhythms with TMS over the FEF to increase visual awareness (Chanes et al., 2013) also open new perspectives for treatment. In addition, MEG experiments in 5 right stroke neglect patients revealed that target omissions correlated with a build-up of low beta activity in left frontal locations before target presentation (Rastelli et al., 2013). Thus, neglect occurrence seems to arise from abnormal oscillatory activity and consequently could be manipulated with rhythmic TMS. Whereas traditional rTMS protocols have shown complex effects on brain oscillations (Thut and Pascual-Leone, 2010; Vernet et al., 2013), the use of rhythmic TMS or Transcranial Alternate Current Stimulation (tACS) combined with EEG demonstrated a possibility to entrain physiologically relevant oscillations at a chosen frequency (Thut et al., 2011; Helfrich et al., 2014). Similarly, the use of double-coil (bifocal) stimulation is a promising tool to modulate synchronization between distant brain areas (Plewnia et al., 2008). Future studies are needed to explore the possibility to induce such modulations in clinical populations lasting long enough to be clinically relevant.

### Performing 3D eye movements to improve visuo-spatial awareness

Another topic of interest for the treatment of visual awareness disorders would consist in further exploring the link between eye movements and conscious perception. Indeed, eye movements training can be natural way to activate the FEF and other areas of attentional networks. The resulting plasticity might in turn improve visual awareness. Vergence is particularly fragile and subject to aging, fatigue and neurological insults (Scheiman et al., 2005a,b,c; Yang et al., 2010) and hence reeducation of eye movements in depth might promote better visual navigation in space and as a consequence improve conscious perception.

The link between exploratory movements performed in 3D space and the gathering of visuo-spatial information goes beyond a simple sharing of brain resources. Indeed, efferent copies of vergence movements, proprioception on convergence state (Priot et al., 2012), or simply disparity indices on which vergence movement can be programmed (Ziegler and Hess, 1997) are all candidates to be involved in our ability to assess depth. Conversely, our understanding of the 3D space can trigger movements in accordance with our perception. However, there are striking examples of dissociations between our perception and action as for instance the famous Müller-Lyer illusion, in which erroneous judgments about an object size and correct manual seizing movement may coexist. This type of experience led to the traditional dissociation between vision for perception and vision for action (Milner and Goodale, 2008). However, such dissociation is controversial and might be an experimental artifact. Indeed, without any visual feedback, hand and eye movements (and consequently the “vision for action”) can also be affected by the illusion (Bruno and Franz, 2009; Bruno et al., 2010). Similarly, in illusions where the perceived depth is different from the actual depth, the vergence movements are sometimes subject to the depth cues of the physical world (Wismeijer et al., 2008), and sometimes to the illusory percept (Sheliga and Miles, 2003). An interesting interpretation is that there are two types of convergence: a fast one to serve action and a slower one, which would allow the construction of a conscious percept (Wagner et al., 2009).

Besides the deficit of awareness in contralesional space, neglect patients seem to also suffer from problems that are specific to the depth at which tasks are performed: neglect symptoms might be sometimes more severe in the near space or in the far space (see Aimola et al., 2012 for a review). Probably, the evaluation paradigms and tasks, the effector used, and the location and extent of the right hemisphere damage could explain the differences found by different studies with regards to this issue (see Aimola et al., 2012 for a discussion). Experimental evidence supports the notion that the representations of peripersonal and extrapersonal spaces are subtended respectively, by rather dorsal or ventral regions within fronto-parietal systems (Aimola et al., 2012). Hence, performance dissociation between tasks performed in near and far space could reveal the specificity of the disconnection patterns between areas required for the task and areas devoted to monitor specific portions of the space (Weiss et al., 2000). In any case, it is possible that neglect patients show deficits in visual navigation in depth, which could aggravate their difficulties in exploring fronto-parallel space for certain depths. Oculomotor training focusing on vergence movements in depth (Jainta et al., 2011) could be another interesting path to explore for a more effective rehabilitation of awareness disorders.

## Conclusion

In this review, we focused on the role and localization of the human FEF, a cortical area which is part of highly distributed saccadic and visuo-spatial networks with important bearing on the control of eye movements in 3D space and contributing importantly to several aspects of attentional and visual cognition. A particular emphasis was placed on TMS studies, which have allowed a successful causal exploration of the contributions of this frontal region. We provided evidence that the results of such studies with regards to their ability to map FEF cortical location are not necessarily similar to those of other mapping techniques employed in neuroscience, such as lesion studies, microstimulation, intracranial recordings, PET, fMRI, and EEG/MEG in both humans and non-human primates. Similarities and discrepancies across the results provided by different techniques, the use of different paradigms and/or experimental models were presented and discussed. Finally, we speculated on the posibility to manipulate FEF activity with non-invasive neurostimulation and oculomotor training in order to improve visuo-spatial awareness in 3D space in healthy population and to promote functional recovery in stroke patients.

### Conflict of interest statement

The authors declare that the research was conducted in the absence of any commercial or financial relationships that could be construed as a potential conflict of interest.

## Abbreviations

CEF: cingulate eye field
DLPFC: dorsolateral prefrontal cortex
EEG: electroencephalography
FEF: frontal eye field
fMRI: functional magnetic resonance imaging
IPS: intraparietal sulcus
LIP: lateral intraparietal area
MEG: magneto-encephalography
MST: medial superior temporal
PEF: parietal eye field
PET: positron emission tomography
PPC: posterior parietal cortex
rTMS: repetitive transcranial magnetic stimulation
SC: superior colliculus
SEF: supplementary eye field
tACS: transcranial alternate current stimulation
tDCS: transcranial direct current stimulation
TMS: transcranial magnetic stimulation.
