# Corrigendum: Frontal eye field, where art thou? Anatomy, function, and non-invasive manipulation of frontal regions involved in eye movements and associated cognitive operations

**DOI:** 10.3389/fnint.2014.00088

**Published:** 2014-11-11

**Authors:** Marine Vernet, Romain Quentin, Lorena Chanes, Andres Mitsumasu, Antoni Valero-Cabre

**Affiliations:** Centre National de la Recherche Scientifique, Institut du Cerveau et de la Moelle EpinièreParis, France

**Keywords:** FEF, brain mapping, transcranial magnetic stimulation, visual performance, visuo-spatial attention, 3D vision, visual awareness, plasticity rehabilitation

A few errors were introduced during the proof reading process, which the authors wish to correct with this corrigendum.

**A few sentences may lead to erroneous interpretations and should be reformulated**

**Page 5, line 3**

Incorrect: *distinct functions related to saccadic activity*.
Correct: *distinct functions related to eye movements*.

**Page 10, column “Interpretation,” square 10**

Incorrect: *Interference with the programming and the execution of saccades (including perceptual analysis of the go signal)*
Correct: *Interference with one or several stages of the programming and the execution of saccades (including perceptual analysis of the go signal)*

**Page 14, column “Effects,” square 2**

Incorrect: *contralaterally (i.e., for left targets) after left FEF stimulation*
Correct: *contralaterally (i.e., for right targets) after left FEF stimulation*

**Page 15, column 2, second paragraph, line 3**

Incorrect: *several studies in healthy humans, combining TMS with EEG Taylor et al., (2007), TMS with fMRI (Ruff et al., 2006) or employing double coil TMS and psychophysics*
Correct: *several studies in healthy humans, combining psychophysics with TMS and EEG Taylor et al., (2007), TMS and fMRI (Ruff et al., 2006) or double-coil TMS (Silvanto et al., 2006)*

**Page 15, last paragraph, line 1:**

Incorrect: *In line with animal and human studies showing respectively, enhanced perception and increased activity in visual areas following FEF stimulation*
Correct: *In line with animal and human studies showing enhanced perception and increased activity in visual areas following FEF stimulation*

**A few typos, grammatical mistakes and erroneous extra-words should be removed from the manuscript**

**Page 2, Part “Localization of FEF,” line 20:**

Incorrect: *Overall, it is still not entirely clear whether the reported inter-species differences in FEF location can be related to genuine anatomical differences between non-human primates and humans, caused by the use of different mapping methods or they simply reflect interindividual differences*
Correct: *Overall, it is still not entirely clear whether the reported inter-species differences in FEF location can be related to genuine anatomical differences between non-human primates and humans, or caused by the use of different mapping methods, or whether they simply reflect interindividual differences*

**Page 8, column 2, line 1**

Incorrect: *The general pictures emerging from this literature is […]*
Correct: *The general picture emerging from this literature is […]*

**Page 11, last paragraph, lines 4 and 6**

Incorrect: *most of the effects on latency modulations*
Correct: *most of the effects on latency*
Incorrect: *anti-saccades modulation*
Correct: *anti-saccades task*

**Page 13, column “Effects,” square 7:**

Incorrect: *TMS stimulation*
Correct: *TMS*

**Page 14, 3 lines before the end:**

Incorrect: *These authors showed that a decrease of the visual sensitivity explained by […]*
Correct: *These authors showed a decrease of the visual sensitivity, explained by […]*

**Page 15, column 2, paragraph 2, line 11**

Incorrect: *Similar short 5-pulse trains of 9 Hz TMS over the right FEF modulated the BOLD activity recorded with fMRI within visual areas V1-V4 led to activity increases […]*
Correct: *Similar short 5-pulse trains of 9 Hz TMS over the right FEF modulated the BOLD activity recorded with fMRI within visual areas V1-V4, leading to activity increases […]*

**Page 16, last paragraph, line 3**

Incorrect: *This hypothesis has been confirmed by the study by Smith et al. (2005), that in agreement with this notion, showed that in a visual detection task, […]*
Correct: *This hypothesis has been confirmed by the study by Smith et al. (2005), showed that in a visual detection task, […]*

**Page 16, last paragraph, line 10**

Incorrect: *Such disruption of the inhibition for unattended locations could also explain the results reported by Ro et al. (2003) who showed that single TMS pulses, delivered over the right FEF, showing that single 600 ms after the cue and 150 ms prior to target onset, decreased the inhibition of return phenomenon*.
Correct: *Such disruption of the inhibition for unattended locations could also explain the results reported by Ro et al. (2003), who showed that single TMS pulses, delivered over the right FEF 600 ms after the cue and 150 ms prior to target onset, decreased the inhibition of return phenomenon*.

## Conflict of interest statement

The authors declare that the research was conducted in the absence of any commercial or financial relationships that could be construed as a potential conflict of interest.
